# ‘Around the edges’: using behaviour change techniques to characterise a multilevel implementation strategy for a fall prevention programme

**DOI:** 10.1186/s13012-018-0798-6

**Published:** 2018-08-20

**Authors:** S. McHugh, C. Sinnott, E. Racine, S. Timmons, M. Byrne, P. M. Kearney

**Affiliations:** 1 School of Public, University College Cork, Cork, Ireland; 2 THIS Institute (The Healthcare Improvement Studies Institute), University of Cambridge, Cambridge, UK; 3 Center for Gerontology and Rehabilitation, University College Cork, Cork, Ireland; 4 Health Behaviour Change Research Group (HBCRG), School of Psychology, National University of Ireland Galway, Galway, Ireland

**Keywords:** Implementation, Intervention content, Behaviour change, Fall prevention, Qualitative

## Abstract

**Background:**

Implementation strategies are needed to ensure that evidence-based healthcare interventions are adopted successfully. However, strategies are generally poorly described and those used in everyday practice are seldom reported formally or fully understood. Characterising the active ingredients of existing strategies is necessary to test and refine implementation. We examined whether an implementation strategy, delivered across multiple settings targeting different stakeholders to support a fall prevention programme, could be characterised using the Behaviour Change Technique (BCT) Taxonomy.

**Methods:**

Data sources included project plans, promotional material, interviews with a purposive sample of stakeholders involved in the strategy’s design and delivery and observations of staff training and information meetings. Data were analysed using TIDieR to describe the strategy and determine the levels at which it operated (organisational, professional, patient). The BCT Taxonomy identified BCTs which were mapped to intervention functions. Data were coded by three researchers and finalised through consensus.

**Results:**

We analysed 22 documents, 6 interviews and 4 observation sessions. Overall, 21 out a possible 93 BCTs were identified across the three levels. At an organisational level, identifiable techniques tended to be broadly defined; the most common BCT was *restructuring the social environment*. While some activities were intended to encourage implementation, they did not have an immediate behavioural target and could not be coded using BCTs.

The largest number and variety of BCTs were used at the professional level to target the multidisciplinary teams delivering the programme and professionals referring to the programme. The main BCTs targeting the multidisciplinary team were *instruction on how to perform the (assessment)* behaviour and *demonstration of (assessment) behaviour*; the main BCT targeting referrers was *adding objects to the environment*. At the patient level, few BCTs were used to target attendance.

**Conclusion:**

In this study, several behaviour change techniques were evident at the individual professional level; however, fewer techniques were identifiable at an organisational level. The BCT Taxonomy was useful for describing components of a multilevel implementation strategy that specifically target behaviour change. To fully and completely describe an implementation strategy, including components that involve organisational or systems level change, other frameworks may be needed.

**Electronic supplementary material:**

The online version of this article (10.1186/s13012-018-0798-6) contains supplementary material, which is available to authorized users.

## Background

In a system as complex as healthcare, change does not happen by itself [1]. Implementation strategies are increasingly recognised as essential for realising the full benefits of evidence-based healthcare, and many have been shown to be effective in changing clinical practice [2, 3]. An implementation strategy refers to how a clinical, public health or health service intervention is implemented [4]. An implementation strategy is typically a broad ‘package’ of techniques wrapped around, and often obscured by, the intervention designed to produce health outcomes [5]. Distinguishing between an intervention and its implementation strategy is an important first step in testing the efficacy of strategies and selecting appropriate outcomes to assess implementation success [6].

Almost 10 years ago, Michie and colleagues highlighted the poor and inconsistent description of complex behaviour change interventions [7]; terms were used interchangeably and the development and content of interventions was rarely driven by an explicit theory of change. The same weaknesses have been highlighted in the design and reporting of implementation strategies: inconsistent use of terminology, lack of conceptual clarity and underutilisation of theory to select strategies [8]. Outside of research, the process for selecting the content of an implementation strategy in the health system is also likely to be unsystematic and atheoretical. For policy makers, managers and healthcare professionals (HCPs) tasked with introducing a clinical intervention, there is a potentially confusing array of options [2]. Decisions are influenced by the type of intervention being implemented and the context in which it is being introduced [9], and health systems are increasingly constrained by competing demands for attention and limited resources to make change possible [10]. Also, strategies may be selected to address expected barriers based on previous experience, rather than targeting the most salient barriers that have been identified systematically in advance. In an analysis of quality improvement studies, Bosch and colleagues found that the reasons for believing a particular strategy would overcome a particular barrier to change were often not explained [11].

Tools and checklists, such as the Template for Intervention Description and Replication (TIDieR), have been developed to guide the development and to standardise reporting of complex interventions [5, 12–15]. The TIDieR framework describes the main features of an intervention and promotes the reporting of any activities to enable or support an intervention [15]. Complementary tools have been developed in behavioural science, which describe intervention and implementation activities in even greater detail using an agreed standardised terminology [14, 16]. One of the most commonly applied frameworks for defining activities in behaviour change interventions is the Behaviour Change Technique (BCT) Taxonomy [16]. The Taxonomy contains 93 discrete behaviour change techniques (BCTs) considered to be the ‘active ingredients’ of an intervention. A BCT is defined as an ‘observable, replicable and irreducible component of an intervention’ that has the potential to change behaviour [16]. BCTs serve different and often multiple intervention functions.

The original development of BCT Taxonomy drew on six classification systems of techniques used primarily to change lifestyle behaviour, and the developers highlighted the importance of examining the extent to which the taxonomy is generalizable across behaviours, disciplines and countries [16]. It has been argued that successful implementation requires individuals at multiple levels to do things differently [17]. Yet, the behaviours that support implementation at the organisational level are often overlooked or are poorly described. While individual organisational theories focus on different organisational factors and how they influence the implementation processes [18], the BCT Taxonomy potentially provides a way to systematically and comprehensively describe the behavioural aspects of these processes at multiple levels, using an agreed terminology. The extent to which the taxonomy can be used to characterise the activities within a complex implementation strategy, operating at multiple levels of the health system and targeting a range of stakeholders, is less well understood. Few studies have applied the BCT Taxonomy to retrospectively characterise implementation strategies. Presseau et al. analysed implementation interventions for diabetes prevention within the confines of randomised controlled trials [17]; Steinmo et al. characterised an implementation strategy used for a discrete clinical intervention in an acute care setting [19]. In the latter example, healthcare professionals were the sole targets and the implementation strategy was delivered in a single setting.

We examined the utility of the BCT Taxonomy to retrospectively characterise the content of an implementation strategy that was developed pragmatically (without reference to theory), spans several settings and targets multiple stakeholder groups. The strategy is being used to implement a fall risk assessment programme for older people, an example of an evidence-based intervention that has been the subject of over 100 intervention trials [20] (see Additional file 1). However, there has been suboptimal translation of this evidence into practice [20]. By describing the implementation strategy in detail using a standardised framework and terminology, we can move towards testing effectiveness and replication in other settings [5, 19, 21]. By retrospectively characterising an existing implementation strategy, we will also gain a better understanding of ‘implementation as usual’ in the health system and identify targets for improvement [22].

## Methods

We conducted a qualitative analysis, using multiple sources of data, to build a comprehensive description of the implementation strategy being used in the fall prevention programme delivered in the Health Service Executive (HSE) in Cork city and county (Republic of Ireland). Data were gathered concurrently over a 6-month period (April–September 2016) (see Additional file 2).

### Document analysis

Documents describing the fall risk assessment clinics or their implementation were provided by the implementation steering group. This multidisciplinary group of health professionals and health service managers were responsible for planning, managing and overseeing implementation of clinics. We also collected documents relating to the broader fall prevention pathway, promotional material, referral forms and documents used by staff during the fall risk assessment clinic (e.g. risk assessment tool) (see Additional file 3 for an outline of the documents included).

### Interviews

Semi-structured face-to-face interviews were conducted with a purposive sample from the 12-member implementation steering group. We followed the principles of critical case sampling to select participants who would yield the most information about day-to-day implementation [23]. As our aim was to examine whether the content of an implementation strategy could be characterised using the BCT Taxonomy as opposed to evaluating its acceptability among the target population or its impact on behaviour change, we did not include interviews with staff and potential referrers in this analysis. Potential participants were invited via email, telephone or in person and were provided with an information sheet outlining the study objectives. As the research lead on the steering group, the lead researcher (SMH) was known to participants.

During interviews, participants were asked to describe what they did to support the set up and running of the fall risk assessment clinics. The interview topic guide was informed by TIDieR reporting tool for complex interventions [15] and included probes on the implementation processes, activities, their purpose and timing. Prompts were added to the topic guide, informed by concurrent observation and document analysis. Interviews were digitally recorded and transcribed verbatim (ER).

### Observation

One researcher (SMH) conducted unstructured observations of training sessions with multidisciplinary teams (MDTs) delivering fall risk assessment clinics and meetings with health professionals eligible to refer to the clinics [24]. As the timing of sessions was determined by those delivering and receiving the training a convenience sample of interactions was selected. Observations took place at two primary care sites where training and the risk assessment clinics were being delivered (April–June 2016). The researcher, who previously conducted interviews with team members, adopted a ‘complete observer’ role [25], attending with the explicit purpose of collecting data and did not participate in the sessions.

Observation notes were taken to record the implementers’ actions and health professionals’ behaviours and responses in context. Notes were written up in full at the end of each period of observation.

### Analysis

All data sources (documents, interview transcripts and observation notes) were imported into NVivo software to support data management and analysis. Interviews and observation notes were anonymised prior to analysis and reporting.

Data analysis was guided by the principles of directed content analysis, a deductive approach in which coding can begin with predetermined codes [26]. Initially, data were coded deductively (by SMH, trained in health services research) using the TIDieR framework which helped to clarify the underlying rationale for the implementation strategy [15]. Where possible, we also coded the population and behaviour being targeted [21]. Data were coded to identify what was carried out, at what level (organisational, professional, patient), by whom, how, where, when and how much and any planned tailoring or modification during the study period.

Data were then coded by one researcher (SMH) using the BCT Taxonomy (version 1) of 93 techniques which are organised into 16 categories. First, data were coded to label potential BCTs and these segments of text were checked carefully against BCT definitions to determine whether a BCT was present or absent [16]. Once this coding was complete, each BCT definition in the taxonomy was reviewed and the researcher (SMH) assessed all three data sources for presence/absence of this BCT. Implementation activities that did not fit with a BCT taxonomy description were retained and coded using in vivo codes (words and phrases used by participants in the data).

A selection of the data was coded by two other researchers, one familiar with the service through a wider evaluation (ER—trained in public policy) and one who was independent of the project (CS—primary care physician and clinical researcher). Each researcher coded a document, field notes from one observation and an interview transcript deemed (by SMH) to include the richest description of the implementation strategy. All coders had participated in online BCT training and had experience coding qualitative data. Coding discrepancies were listed (by SMH) and reviewed by the coding team (CS, ER). Researchers outlined their rationale for coding a BCT, and the group reviewed the clarity and volume of data available to support its presence. An accumulation of segments of text led to greater certainty about the presence or absence of a BCT rather than a single line of text, so when an interview transcript was taken in its entirety, researchers tended to code the same BCTs. In a small number of cases where researchers identified different BCTs, this was typically due to different interpretations of the target or purpose of the implementation activity. These discrepancies were reviewed together and resolved by discussing our individual coding rationale, consulting other sources of data and drawing on the lead researcher’s knowledge of the implementation context. Outstanding discrepancies and uncertainties were reviewed by a behavioural scientist with experience in the use of BCTs (MB). Any resultant changes to coding were applied across the data.

BCTs were mapped to intervention functions, guided by data on the rationale and purpose of the implementation strategy and links between functions and BCTs outlined in the *Behaviour Change Wheel Guide to Designing Interventions* [27].

Sampling, data collection and analysis were iterative. As initial data were analysed, participants were recruited using the same sampling and contact methods described above and additional documents were sought to elaborate on specific aspects of the implementation strategy. We purposively chose a sample of critical cases, interactions and documents that provided the best opportunity to gather sufficient depth of information to fully describe the implementation strategy being studied [28]. The stages of analysis and codes were revisited multiple times. We assessed construct saturation, whether the conceptual categories of the BCT Taxonomy and intervention functions were adequately populated, based on the analysis of all data sources in consultation with the definitions in the BCT Taxonomy.

### Coding assumptions

We made a number of assumptions during analysis. These assumptions were adapted from the approach used by Presseau et al. to code multilevel implementation interventions [29]. First, we focused on techniques used to support or enable the *implementation* of the fall risk assessment clinics (implementation strategy), as distinct from techniques used during the risk assessment itself (clinical intervention).

Second, in line with the premise of the Behaviour Change Wheel approach, we assumed that the implementation of an intervention depends on behaviour change at some point [12]. We assumed that all BCTs (included those at the system level) functioned by targeting the behaviour of health service managers, HCPs (referring to and/or running clinics) and patients, to ultimately influence implementation outcomes.

Third, there were a range of target behaviours which were broadly defined as ‘implementation behaviours’. The specific behaviour varied depending on the actor and level of the strategy being considered. In some cases, the target behaviour was clear and thus BCTs were coded at that level (e.g. referring clients to the clinic). Where the specific target behaviour was not clear, BCTs were coded at a more general level of ‘supporting the implementation of the fall risk assessment clinics’.

Fourth, we considered a BCT to be present based on an accumulation of text segments across a single source of data or multiple sources (document, interview and/or observation field notes). If it was unclear whether a BCT was present or absent, following discussion and consideration of multiple data sources, it was not coded. We did not apply a minimum frequency of occurrence of BCTs; techniques that occurred once were coded. Fifth, planning and initial implementation occurred in tandem with data collection. Therefore, some BCTs were planned while others had taken place at the time of data collection.

The Standards for Reporting Qualitative Research (SRQR) was used to guide the reporting of findings (see Additional file 4) [30].

## Results

Twenty-two documents, six interviews and four observations were conducted across two implementation sites (see Additional file 3).

### Components of the implementation strategy and target behaviours

Table 1 presents the main components of the implementation strategy, stratified by target level (i.e. organisational, professional or patient level) [15]. In summary, at an organisational level, the implementation strategy involved establishing a multidisciplinary steering group led by a clinical project manager, appointing an implementation coordinator and administrator and assembling multidisciplinary teams to deliver clinics. Multiple behaviours were targeted simultaneously at this level making it difficult to identify a specific behavioural target; thus, the target behaviour was defined broadly as ‘supporting implementation of fall risk assessment clinics’.

**Table 1:** Components of the implementation strategy described using the TIDieR framework [15]

	What	Why	Who		Target behaviour	How, when and how often	Tailoring	Modifications
	Component	Rationale	Delivered by	Delivered to		Mode and frequency	Planned adaptation	During the study
Organisational level	1. Implementation steering group led by clinical project manager	To ensure ‘successful planning, execution, monitoring, controlling and closing of the project’ (document). Project manager ‘problem solving’ (I)	Project Manager, coordinator, representative from hospital, community, management	Heads of disciplines, management, MDTs, referrers	Multiple behaviours: ‘supporting implementation of fall risk assessment clinics’	Face to face monthly meetings, ongoing email and telephone contact	‘Communication tailored to the requirements of different audiences’ (D)	
	2. Appointed coordinator and administrator	To create ‘single point of contact’ for referrers, MDTs and clients. Previous efforts failed due to lack of ‘practical support’ (I)	NA	MDTs, referrers, heads of discipline, management	Multiple behaviours: ‘supporting implementation of fall risk assessment clinics’	Ongoing meetings, phone and email contact with MDTs and referrers	Mode of communication *‘depends on the person’* in each clinic (I)	
	3. Set up MDT to deliver assessment	Identify and assemble team of physiotherapist, occupational therapist, nurse	Coordinator Project manager	1. Head of discipline 2. Line managers	Multiple behaviours: ‘supporting implementation of fall risk assessment clinics’	Face to face meetings and phone contact prior to initiating clinic	No reference	
Professional: multidisciplinary team	4. Training and ‘coaching’	To provide *‘coaching and mentoring to MDTs*’ in conducting assessment to ensure team were *‘comfortable’*. (I)	Coordinator Administrator Specialist fall team	MDT	Delivering risk assessment clinic	Face to face Prior to initiation and during weeks 2–3 of implementation	No reference	Number, timing and duration varied based on knowledge, requests and availability
	5. Standard assessment form	Enable standardised assessment and onward referral	Coordinator Administrator	MDT	Delivering risk assessment clinic	Circulated prior to initiating clinic	No reference	Format and level of information changed during pilot
	6. Equipment	To ensure assessment could be conducted	Coordinator Administrator	MDT	Delivering risk assessment clinic	Prior to initiating clinic	Dependent on existing equipment	
Professional: referrers	7. Standard referral form	Enable efficient referral to service	Coordinator	Referrers	Refer to clinic	Circulated during initial implement	No reference	Level of information changed during pilot
	8. Information meetings with referrers	‘Selling’ clinics to get referrers ‘on board’ and *‘to discuss criteria on who we want (referred) and [ensure] that is very clear’.(I)*	Coordinator Specialists Project manager	Physicians ANPs PHNs	Refer to clinic	Ad-hoc face to face meetings ‘ideally’ before clinic started (I)	Timing depended on clinic being established in that area	Number of meetings increased in areas with low referral rates
	9. Screening tool for PHNs	Generate referrals for the clinics among PHNs who ‘*would be the first line of contact with the health service.’ (I)*	Coordinator Director of Public Health Nursing	PHNs	Identify eligible clients and refer to clinic	Ad-hoc face to face meetings to introduce and promote use of tool	No reference	Number and timing of meetings varied by area and level of engagement
	10. Promotional material	Advertise and inform referrers about clinics	Coordinator Administrator	Referrers Pharmacies, Day centres	Refer to clinic	Flyers, posters, monthly mail shot (to GPs)	No reference	
Patient	11. Invitation letter and information leaflet	To inform clients about appointment, clinic location and how to prepare for their visit, centralising administration to support MDTs.	Coordinator Administrator	Clients	Attend clinic	Documents provided on receipt of referral and arrangement of appointment	No reference	

*Admin* administrator, *ANP* advanced nurse practitioner, *Ax* assessment, *Comms* communication, *D* document, *I* interview, *IC* implementation coordinator, *MDT* multidisciplinary team, *Mgmt* management, *PHN* public health nurse, *PM* project manager, *SG* steering group

Most of the implementation strategy targeted either (1) the behaviour of professionals in the multi-disciplinary team responsible for ‘delivering the fall risk assessment clinic’ or (2) the behaviour of professionals ‘referring to the clinic’ (including primary care physicians, public health nurses, physiotherapists, occupational therapists and advanced nurse practitioners in emergency departments). At this level, the strategy focused on training and ongoing ‘coaching’ for multidisciplinary teams, and standardised referral documentation and promotional meetings with referrers. At the patient level, a standardised invitation letter and information leaflet were developed to encourage referred patients to ‘attend the clinic’.

The implementation strategy was tailored depending on existing resources at each site, and communication was ‘tailored to the requirements of different audiences’. Other components were modified during initial implementation in response to the needs identified at each site, for example the number of training sessions varied according to the health professional and his/her experience.

### Behaviour Change Techniques (BCTs)

Overall, 21 out a possible 93 BCTs, from 14 of the 16 categories in the taxonomy, were identified (indicated in italics). Table 2 illustrates the BCTs used at each level of the implementation strategy (Additional file 5 describes each BCT identified within each component, with supporting data). The most commonly used BCTs were *instruction on how to perform the behaviour* and *adding objects to the environment*.

**Table 2:**
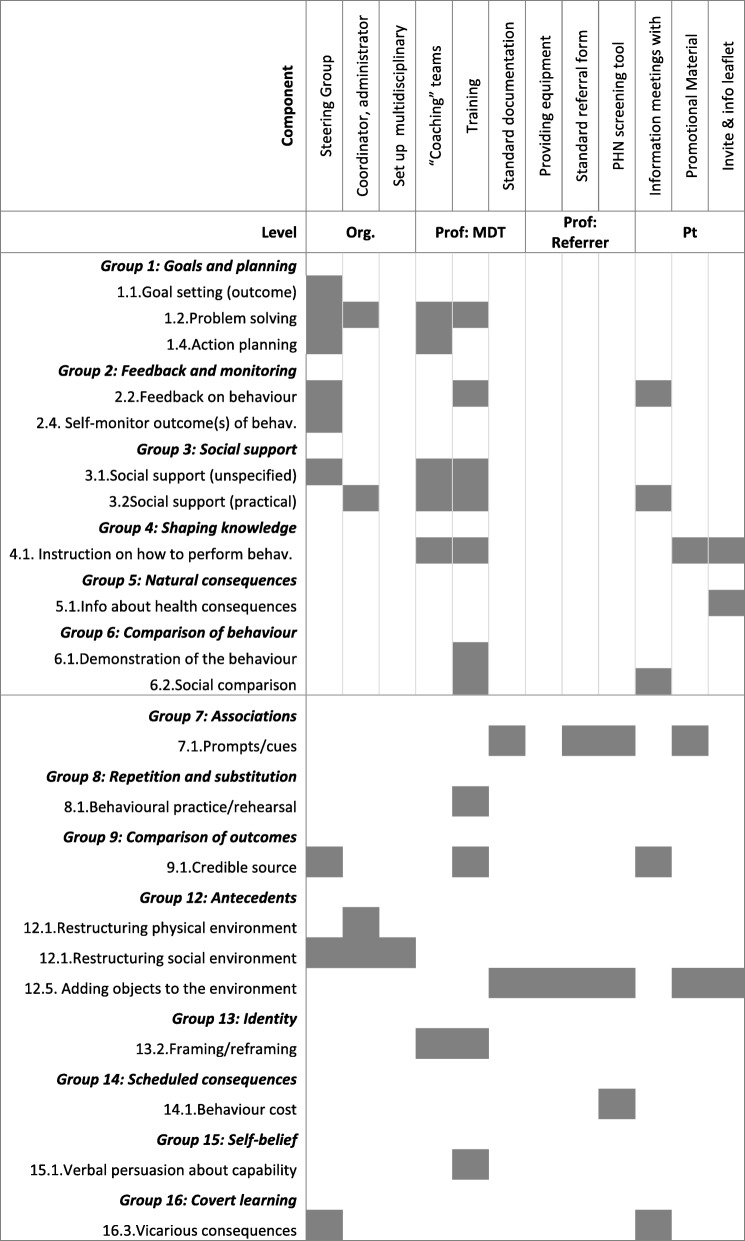
Map of BCTs identified across levels and components of the implementation strategy

*Org* organisational level, *Prof* professional level, *Pt* patient/client level, *PHN* public health nurse

### BCTs and functions used at each level of the implementation strategy

#### Organisational level

At the organisational level, the target behaviour was defined as ‘supporting the implementation of fall risk assessment clinics’. The steering group’s activities concentrated ‘around the edges [of the programme], problem solving and making sure that it’s presented well’ (interview). Overall, 11 BCTs were coded from six of the 16 categorises. The main BCT used was *restructuring the social environment*.

Even within the organisational level, multiple layers of BCTs were identifiable (see Additional file 5). For example, we coded the establishment of the implementation steering group itself as restructuring the social environment but there were also specific BCTs evident within the work of the steering group such as ‘monitoring and analysing pathway flows and activity, [to] adapt and refine the [fall prevention] pathway’ (document). This activity was coded as the steering group *self-monitoring outcome(s) of behaviour* to change their own implementation activities. Similarly, members of the steering group who were line managers ‘sold’ fall risk assessment clinics to their staff using *framing/reframing* and *vicarious consequences* (for older people):

‘I tell them it's an integrated service for an actual screening tool to be used within the community, and that they're the decision makers around it. They can decide whether the client needs to be referred, they can do the plan of care there and then [and] the client can provide input for what they need.’ (interview)

#### Professional level

We classified target behaviours at the professional level as behaviours relating to ‘delivering the fall risk assessment clinic’ and those relating to ‘referring to the clinic’. The largest number and variety of techniques were identifiable at the professional level. Overall, 16 techniques were coded from 13 of 16 groups of BCTs.

The main BCTs used to target multidisciplinary professionals tasked with ‘delivering the fall risk assessment clinics’ were providing *instruction on how to perform the assessment* and *demonstration of assessment behaviour*, serving a training function. Training was delivered by a *credible source* (implementation coordinator with clinical expertise and experience in falls prevention) and included *verbal persuasion about capability*. Previous efforts to establish fall prevention services had failed because of a lack of ‘practical support’ for health professionals, thus *social support* (unspecified and practical) and *problem solving* were also used.

To support health professionals ‘referring to the clinic’, a smaller number and range of BCTs were used. The most common BCTs were *adding objects to the environment* (standardised referral form) which included *prompts and cues* and using *credible sources* to meet with referrers to persuade them to use the service. Table 3 shows differences in the BCTs used to target the range of HCPs eligible to refer to the service: primary care physicians were told about the vicarious consequences (shorter waiting times) of referring to the single point of access; *social comparison* was used to persuade public health nurses whereby colleagues involved in the delivery of clinics would ‘(anonymously) feedback outcomes of clients…, how this [clinic] works, and how they [the clients] proceeded to the clinic’ (interview); and advanced nurse practitioners would receive *feedback on their referral behaviour*.

**Table 3:**
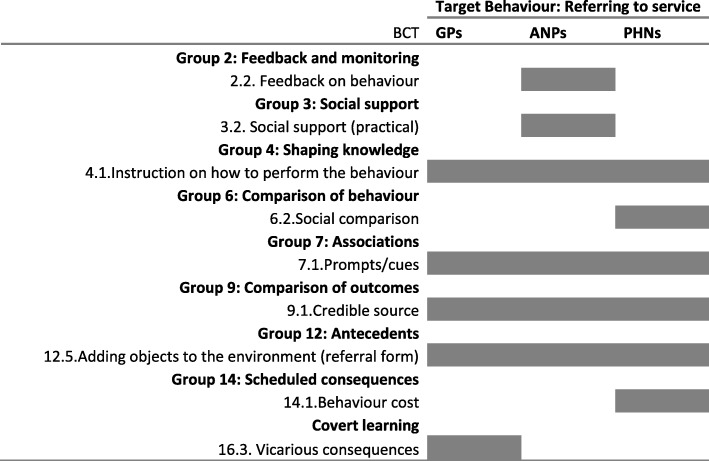
Map of BCTs identified according to target group of referrers

BCTs relating to seven of nine intervention functions were apparent in the implementation strategy. Most BCTs were used to increase the means or reduce barriers to increase capability or opportunity to carry out behaviour (enablement function) (Additional file 5). The two intervention functions not represented by any BCTs were incentivisation and restriction. Only one BCT, *behaviour cost*, served a coercive function, to encourage public health nurses to use a fall risk screening tool to identify eligible clients for referral; ‘any aids or appliances that people are applying for, they won’t be issued unless there’s a falls screen’ (interview).

#### Patient level

At the patient level, the target behaviour was ‘attending the fall risk assessment clinic’ and in total, three techniques were coded from three categories of BCTs. The invitation letter and information leaflet contained *instruction on how to perform the behaviour* (attending the clinic). There was brief *information about the health consequences and social consequences* of attending; the information leaflet indicated that clients ‘may be offered the opportunity to attend a falls education class’ and ‘will be shown how to cope in the event of a fall’.

### Participants’ activities to shape knowledge, attitudes and intentions to act in future

At the organisational level, we found many implementation activities could not be coded using any of the BCTs and that they appeared to target attitudes rather than immediate behaviour change. First, there were activities, described as ‘the hustle’, to generate buy-in and foster goodwill among key stakeholders. These activities appeared to implicitly target stakeholders’ intentions to support clinics in the future for example by releasing staff and space to run clinics (target: heads of discipline) or referring clients (target: health professionals eligible to refer). Selling the clinics served the function of educating health professionals about their ‘existence’ and purpose, but also sought to persuade potential referrers about the benefits and ease of adopting the service.

‘[we tell them] you can’t do without [these clinics] but it’s no trouble [to refer]’ (interview)

Second, piloting activities, which were evaluative and reactive and did not align with goals and planning BCTs such as reviewing behaviour and outcome goals, were used at the first implementation site.

‘What changed was the onward referral form and the process of who we were referring on to, and what form they got and what level of information they got or needed. That changed really on a weekly basis in clinic 1.’ (interview)

## Discussion

### Summary

We examined the utility of the BCT Taxonomy to characterise the content of a real-world implementation strategy targeting a range of stakeholders across organisational boundaries. Overall, 21 distinct BCTs were used to restructure the environment for implementation and to train, educate, enable and persuade health professionals and patients. Of the nine possible intervention functions, none of the techniques sought to restrict behaviour or incentivise behaviour change. While we identified several techniques at the individual professional level, fewer techniques were identifiable at an organisational level. Furthermore, some implementation activities at this level did not target immediate behaviour change. The utility and application of implementation strategies could be improved by adopting a systematic approach using a theory-based framework which identifies active ingredients and offers the potential to understand mechanisms of change [13, 29, 31]. However, to accurately describe and fully understand a multilevel implementation strategy, the behavioural approach may need to be integrated with organisational theories of change.

### Utility of the BCT Taxonomy

Like Steinmo et al., we found that this systematic approach could be applied ‘post hoc’ to an existing implementation strategy that was developed pragmatically based on organisational knowledge and experience. Building on their analysis of an implementation strategy targeting professional behaviour in the hospital setting, our results show that the BCT Taxonomy is also applicable to wider implementation strategies which target multiple groups across different healthcare settings. Similar to Presseau et al., who examined randomised controlled *trials* of diabetes implementation interventions, we found that a small number of the possible BCTs (21/93) were used to support the introduction of a fall prevention programme. The main techniques used to target professional behaviour, including instruction on how to perform behaviour, adding objects to the environment, prompts and cues and social support, were similar across all three studies. The popularity of certain BCTs and the narrow range being used in implementation strategies highlights the potential for practitioners and researchers to think creatively about implementation by drawing on other techniques listed in the BCT Taxonomy [29].

### Characterising organisational-level implementation activities and their targets

In this study, fewer BCTs were identifiable at the organisational level compared to the professional level. Techniques at the organisational level tended to be broadly defined BCTs, such as restructuring the social environment, which were not necessarily specific to the clinical context of fall prevention. Our findings highlight some of the more specific techniques, such as action planning and problem solving, used to operationalise this broad BCT at an organisational level. Rather than simply listing the BCT ‘ingredients’ in an implementation strategy, we believe that techniques should be linked to specific components and levels of delivery in the strategy’s description.

Some activities did not have an immediate behavioural target, highlighting the importance of measuring proximal outcomes (such as attitudes and beliefs) as well as distal outcomes (like changes in behaviour) when evaluating implementation strategies**.** Although the ultimate goal of implementation may be behaviour change, immediate goals include changing organisational determinants of behaviour such as policy and procedures, staffing and resource allocation [32–34]. It could be argued that the recruitment of dedicated staff to coordinate implementation, an example of restructuring the social environment and a resource which enabled most components of the implementation strategy in this study, is targeting organisational as well as individual determinants of change. Research on implementation climate suggests that when organisations align their policies, practices and resources to support implementation, staff have a clearer understanding of the priorities and value placed on an intervention, and thus are more likely to change their behaviour accordingly [35].

### Practical implications

The systematic process of characterising and reporting an implementation strategy may prompt those designing and delivering strategies to think about the intended target(s) and expected outcomes of their activities. Outside of research studies, the process of specifying existing implementation strategies using approaches such as the BCT Taxonomy may be difficult to navigate in practice without support, given the number and granularity of techniques, and the specific terminology. An overarching framework, such as the AIMD (Aims, Ingredients, Mechanism, Delivery), may be a useful and efficient starting point for health care professionals, funders, managers and policy makers seeking to formally describe interventions. This simplified framework is deliberately ‘terminology agnostic’ to promote collaboration across related fields including quality improvement, policy, public health, patient safety and behaviour change [32]. As a meta-framework, it accommodates the use of specific frameworks such as BCT Taxonomy for particular purposes.

Characterising an existing strategy using a well-established framework provides an in-depth understanding of ‘implementation as usual’ in the health system and highlights gaps that can be addressed to refine implementation and maximise effectiveness. For example, in this study, few behaviour change techniques were used to target older peoples’ attendance at the fall prevention programme. Client engagement is a well-established problem for fall prevention programmes; a systematic review of uptake rates in clinical trials of fall prevention interventions reported non-attendance rates as high as 42% [36]. The implementation strategy in this study did not include any monitoring and feedback techniques to target health professionals referring to the service or those health professionals delivering the service. Yet data on referrals and assessment outcomes were collected and analysed to facilitate self-monitoring and action planning among the implementation steering group. Given the availability of valid routine data with mechanisms for audit and feedback from trusted sources within the health service and the substantial evidence of effectiveness for changing professional behaviour [37], there is potential to trial different approaches to and components of audit and feedback within this ongoing organisational initiative [38].

This study identified the behaviour change techniques planned or used during the initial implementation of fall risk assessment clinics in the health service. This is likely only a sample of the techniques that will be used over the course of implementation. Indeed, from the outset, the number and type of behaviour change techniques varied depending on the target group and context; different techniques were used to target referral behaviour among primary care physicians, advanced nurse practitioners in hospitals and public health nurses working in the community. It is widely acknowledged that implementation strategies should be tailored to the local context [39]. Further research is being conducted with the health professionals and clients to explore their experiences of implementation and perceptions of the acceptability and appropriateness of the implementation strategy. We will combine these results with our current understanding of the content and function of the implementation strategy to address gaps or barriers to its success.

### Strengths and limitations

The TIDieR framework helped us to clarify the underlying rationale for the implementation strategy and thus the intended target population for some activities [15]. We supplemented the TIDieR framework with a description of the ‘action targets’ [21], that is population and behaviour being targeted by activities, which was necessary before coding behaviour change techniques.

It was not feasible to observe every interaction, particularly at an organisational level, given the dynamic and often unpredictable nature of implementation and the number of actors and settings involved. Therefore, some BCTs at the organisational level may have been missed. While the number of interviews and observations in this study is small, the underlying sampling rationale was to generate a rich and thick description of the content of an implementation strategy. Thus, our sampling was not driven solely by numbers of interviews but also the appropriateness of the data. A purposively chose sample of participants, interactions and documents provided the best opportunity to gather sufficient depth of information to fully describe the implementation strategy being studied [28, 40]. Given our use of deductive content analysis with a pre-determined framework, we focused on construct saturation over the concept of theoretical saturation from grounded theory. Data collection and sampling was iterative; however, the process of judging saturation may have been facilitated and enhanced by the use of explicit *a prior* stopping criteria which have been used in theory-based interview studies [41]. The identification of BCTs was influenced by the richness of description and the specificity of BCT definitions. As mentioned previously, the definitions of some BCTs are relatively broad which could increase the ease and frequency with which they are coded. Interview participants did not naturally describe their work activities in the language of BCTs. Data from interviews were triangulated with data from documents and observations which provided reassurance about the presence or absence of a BCT [42]. BCTs were typically coded from an accumulation of evidence across data sources rather than relying on a single source.

In line with the Medical Research Council guidance for process evaluations of complex interventions, which warns against forcing an established framework to fit interventions which were not originally designed based on theory [43], we used participants’ own language to code and preserve aspects of the implementation strategy that were not adequately or accurately described using BCT definitions.

In this study, we did not evaluate the performance of behaviour change techniques and therefore cannot comment on the degree to which they were consistently delivered as intended or their impact on behaviour change. Capturing what is delivered in practice is essential for evaluators to identify adaptations that may undermine or enhance effectiveness [43]. Variability in the way professionals deliver an implementation strategy is to be expected as they try to respond appropriately to the needs of the target group and changing context. Hence, systemisation may not always be feasible [44].

## Conclusions

We examined the utility of the BCT Taxonomy for characterising the content of a multilevel implementation strategy used in the ‘real world’ to support an evidence-based fall prevention intervention for older people. Using the BCT Taxonomy, we identified several techniques at the professional level but fewer techniques that constitute implementation behaviour at an organisational level. Some activities did not have an immediate behavioural target thus to fully and accurately describe a multilevel implementation strategy, other organisational frameworks may be needed. Improving the ability of health service managers, clinicians and researchers to firstly consider and, if possible, formalise the content of implementation strategies would facilitate the testing, replication and refinement of implementation strategies, ultimately maximising the value of existing and future clinical-focussed research for healthcare providers and patients.

## Additional files

Additional file 1: Outline of Fall Risk Assessment Clinics. Figure depicting the organisation and main features of fall risk assessment clinics being supported by the implementations strategy. (PDF 172 kb)

Additional file 2: Timeline of initial implementation at two primary care sites and data collection for study. Figure depicting the timeline of initial implementation including the timing of clinics being set up and the implementation strategy being delivered and the overlap with data collection for the current study. (PDF 177 kb)

Additional file 3: Documents collated and analysed. Table of the documents collated and analysed in the study. (PDF 189 kb)

Additional file 4: Standards for Reporting Qualitative Research (SRQR) Checklist. (PDF 328 kb)

Additional file 5: List of BCTs and functions used at the organisational level targeting behaviours ‘supporting the implementation of fall risk assessment clinics’. Table outlining the activities undertaken within each component of the implementation strategy, the BCT coded to that activity and the intervention function(s) it served. (PDF 358 kb)

## Abbreviations

ANP: Advanced nurse practitioner
BCT: Behaviour Change Technique
GP: General practitioner
HCP: Healthcare professional
MDT: Multidisciplinary team
PHN: Public health nurse
TIDieR: Template for Intervention Description and Replication
