# Supplementary figures and images for: ‘Around the edges’: using behaviour change techniques to characterise a multilevel implementation strategy for a fall prevention programme
Source: Implement Sci. 2018 Aug 20;13:113. doi: 10.1186/s13012-018-0798-6 (PMC6102850; doi:10.1186/s13012-018-0798-6)

## Additional File 1

### Outline of Fall Risk Assessment Clinics

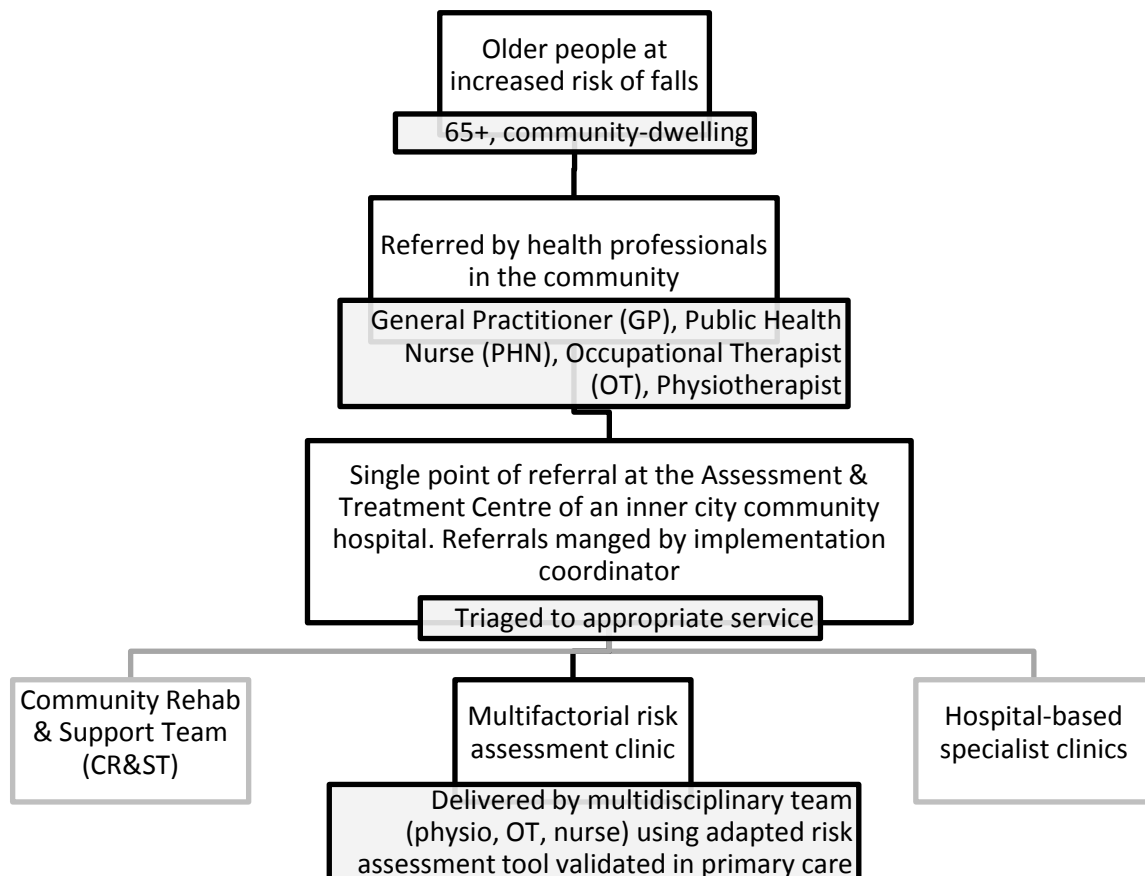

Supplement: Supplementary file 1 — Outline of Fall Risk Assessment Clinics. Figure depicting the organisation and main features of fall risk assessment clinics being supported by the implementations strategy. (PDF 172 kb) [file 13012_2018_798_MOESM1_ESM.pdf]
