# Supplementary material for: ‘Around the edges’: using behaviour change techniques to characterise a multilevel implementation strategy for a fall prevention programme
Source: Implement Sci. 2018 Aug 20;13:113. doi: 10.1186/s13012-018-0798-6 (PMC6102850; doi:10.1186/s13012-018-0798-6)

Additional File 2

Timeline of initial implementation at two primary care sites and data collection for study

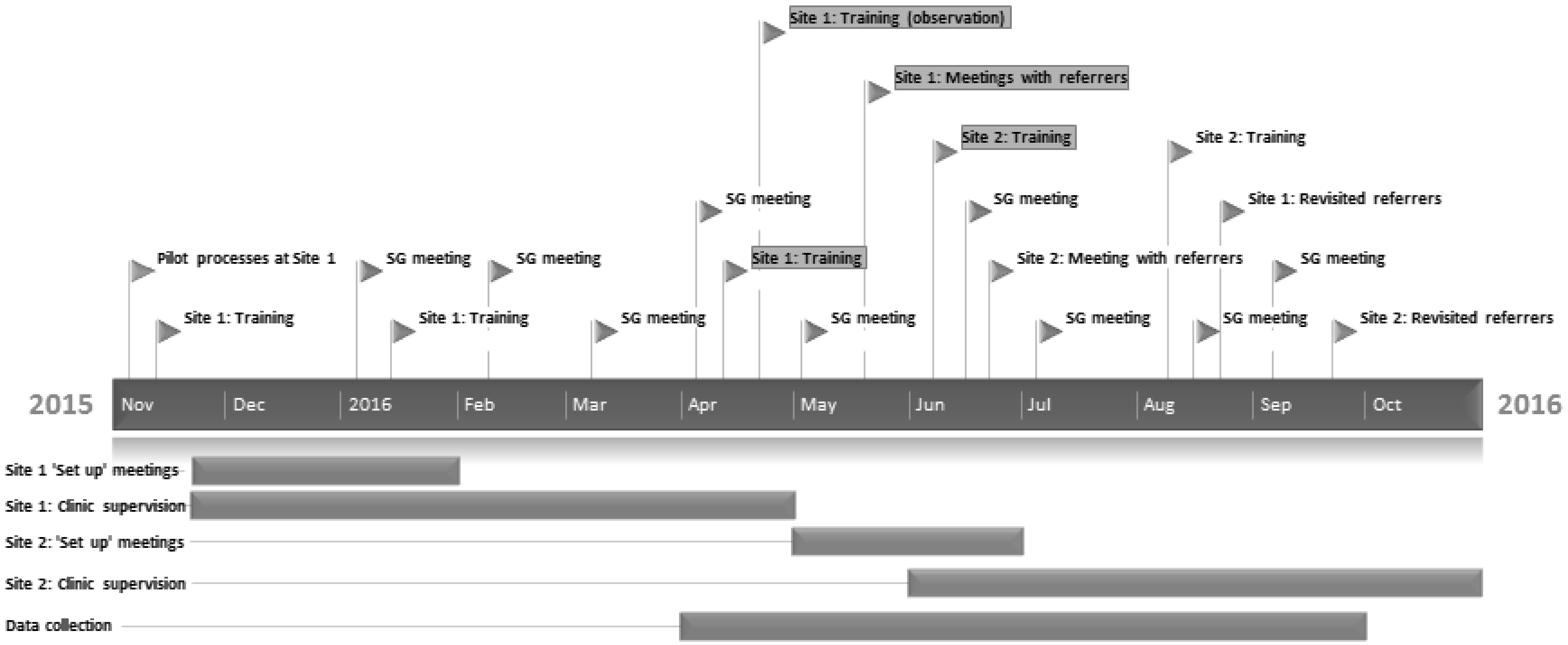

Supplement: Supplementary file 2 — Timeline of initial implementation at two primary care sites and data collection for study. Figure depicting the timeline of initial implementation including the timing of clinics being set up and the implementation strategy being delivered and the overlap with data collection for the current study. (PDF 177 kb) [file 13012_2018_798_MOESM2_ESM.pdf]
