# Supplementary material for: ‘Around the edges’: using behaviour change techniques to characterise a multilevel implementation strategy for a fall prevention programme
Source: Implement Sci. 2018 Aug 20;13:113. doi: 10.1186/s13012-018-0798-6 (PMC6102850; doi:10.1186/s13012-018-0798-6)
Supplement: Supplementary file 3 — Documents collated and analysed. Table of the documents collated and analysed in the study. (PDF 189 kb) [file 13012_2018_798_MOESM3_ESM.pdf]

Additional File 3

Documents collated and analysed

Description	Document	Purpose of the document
**General pathway information**	Project Initiation Document	Prepared by project manager for Health Service Executive to secure funding
	Summary presentation to steering group	Prepared by coordinator and administrator presented to steering group to provide a summary of changes, training and activity
	Job specification for implementation coordinator	Prepared by members of SG before recruiting falls coordinator
	Falls project structure diagram	Overview document setting out SG and terms of reference prepared by project manager
	Meeting minutes	Monthly update on progress to date and actions to be taken during installation and initial implementation at first two clinic sites (Jan-Sept 2016)
**Promotional material**	Flyers	Prepared by communications sub-group for GPs, pharmacies, PHNs, any other source of referral
**Documents used during the multifactorial risk assessment clinic**	Referral form	Prepared by coordinator and administrator for health professionals referring to clinics.
	Client appointment letter	Prepared by coordinator and reviewed by implementation steering group. Sent by administrator to client to notify him/her of appointment at local falls risk assessment clinic.
	Client information leaflet	Prepared by coordinator and administrator, sent to client along with appointment letter.
	'QuickScreen' tool	Prepared by coordinator and administrator for multidisciplinary teams to use during assessment
	Summary of assessment form	Prepared by coordinator and admin for multidisciplinary teams to use on completion of assessment and reviewed by the implementation steering group. This form is returned to central admin and is sent with any onward referrals (e.g. to GP)
	Description of pathway	Prepared by coordinator and administrator to outline core documents used in service & the main processes
	Cover sheet attendance	Prepared by coordinator and administrator for multidisciplinary teams to record input into assessment and activity. Form is returned to central administrator to track activity
	Clinic pathway for Occupational Therapists (OT)	Devised by OTs involved in clinics across sites. Developed in July outlining procedures before, during and after assessment.

Observations

Implementation Strategy Component	Description	Location	Duration
Training	Small group training including instruction on assessment tool, sequence, information required, brief demonstrations of physical assessment, explanation of onward referral process.	Primary care centre, implementation sites 1 & 2	Approx. 2 hours
Training	Observation session during which multidisciplinary team observed the coordinator conducting a risk assessment with a client.	Primary care centre, implementation site 1	Approx. 1 hour for assessment followed by questions and answers.
Meetings with health care professionals eligible to refer to the service	Meetings between clinical members of the implementation steering group (n=3) and primary care physicians (n=6) to introduce service, referral criteria and standardised form.	General Practice	Approx. 30 minutes, after surgery hours
