# Supplementary material for: ‘Around the edges’: using behaviour change techniques to characterise a multilevel implementation strategy for a fall prevention programme
Source: Implement Sci. 2018 Aug 20;13:113. doi: 10.1186/s13012-018-0798-6 (PMC6102850; doi:10.1186/s13012-018-0798-6)
Supplement: Supplementary file 4 — Standards for Reporting Qualitative Research (SRQR) Checklist. (PDF 328 kb) [file 13012_2018_798_MOESM4_ESM.pdf]

Standards for Reporting Qualitative Research (SRQR)		Page/line no(s).
**Title and abstract**		
**S1**	**Title** - Concise description of the nature and topic of the study Identifying the study as qualitative or indicating the approach (e.g., ethnography, grounded theory) or data collection methods (e.g., interview, focus group) is recommended	P1, title indicates the descriptive nature of the study and the topic (using the BCT taxonomy to characterise an implementation strategy)<br><br>Key words include 'qualitative'
**S2**	**Abstract** - Summary of key elements of the study using the abstract format of the intended publication; typically includes background, purpose, methods, results, and conclusions	P2/3: Abstract includes background, purpose of the study, summary of qualitative methods, results and conclusions.
**Introduction**		
**S3**	**Problem formulation** - Description and significance of the problem/phenomenon studied; review of relevant theory and empirical work; problem statement	P4: Significance of the problem of poor and inconsistent description,<br>P4/5 Relevant theory [BCT Taxonomy]<br>P5: Limited empirical work using taxonomy for retrospective specification of implementation.
**S4**	**Purpose or research questio**n - Purpose of the study and specific objectives or questions	P5: Purpose and specific objective to examine the utility of the BCT Taxonomy to retrospectively characterise a multilevel implementation strategy
**Methods**		
**S5**	**Qualitative approach and research paradigm** - Qualitative approach (e.g., ethnography, grounded theory, case study, phenomenology, narrative research) and guiding theory if appropriate; identifying the research paradigm (e.g., postpositivist, constructivist/ interpretivist) is also recommended; rationale**	P6: Qualitative using, using multiple sources of data (documents, interviews, observations).<br>P7/8: The BCT taxonomy was the guiding psychological theory-based framework.
**S6**	**Researcher characteristics and reflexivity** - Researchers' characteristics that may influence the research, including personal attributes, qualifications/experience, relationship with participants, assumptions, and/or presuppositions; potential or actual interaction between researchers' characteristics and the research questions, approach, methods, results, and/or transferability	P6/7: Relationship with participants who were known to lead researcher collecting data<br>P7/8: Researchers' qualifications, training in BCT coding, familiarity with topic and experience in qualitative coding outlined
**S7**	**Context** - Setting/site and salient contextual factors; rationale**	P6: Implementation strategy being used in the fall prevention service of the Health Service Executive (HSE) in Cork city and county (Republic of Ireland).<br><br>Additional file 1 and 2 also include contextual information on the implementation strategy and the study
**S8**	**Sampling strategy** - How and why research participants, documents, or events were selected; criteria for deciding when no further sampling was necessary (e.g., sampling saturation); rationale**	P6/7: outline the selection of documents analysed, purposive sampling strategy for interview participants and convenience sample of observations.<br>Additional file 3 provides detailed information

		about the documents and observations.<br><br>P7: outlines the iterative nature of sampling, data collection and analysis to achieve saturation.
**S9**	**Ethical issues pertaining to human subjects** - Documentation of approval by an appropriate ethics review board and participant consent, or explanation for lack thereof; other confidentiality and data security issues	P19 outlines ethical approval and consent
**S10**	**Data collection methods** - Types of data collected; details of data collection procedures including (as appropriate) start and stop dates of data collection and analysis, iterative process, triangulation of sources/methods, and modification of procedures in response to evolving study findings; rationale**	P6/7: data collection methods including document analysis, interviews and observations.<br><br>P6: outlines detailed 6 month timescale of data collection. Additional file 2 provides further detail<br><br>P6: outlines how topic guide was adapted in response to preliminary analysis across data sources<br><br>P6/8: outline integration and triangulation of data sources
**S11**	**Data collection instruments and technologies** - Description of instruments (e.g., interview guides, questionnaires) and devices (e.g., audio recorders) used for data collection; if/how the instrument(s) changed over the course of the study	P6: describes topic guide informed by TIDieR framework and digital recording<br>P7: field notes from observations described
**S12**	**Units of study** - Number and relevant characteristics of participants, documents, or events included in the study; level of participation (could be reported in results)	P10: number of documents, participants and observations reported. Characteristics of participants not reported due to critical case sampling and potential risk to anonymity of participants.
**S13**	**Data processing** - Methods for processing data prior to and during analysis, including transcription, data entry, data management and security, verification of data integrity, data coding, and anonymization/de-identification of excerpts	P7: data transcription and use of Nvivo software to process, enter and manage data.<br>P7/8: data coding and verification of data integrity involving multiple researchers, anonymization of data
**S14**	**Data analysis** - Process by which inferences, themes, etc., were identified and developed, including the researchers involved in data analysis; usually references a specific paradigm or approach; rationale**	P7/8: outlines phases of analysis and approach taken during each phase, how codes were identified and finalized using BCT taxonomy, role of researchers at each phase.<br><br>P4/5 of introduction outline rationale for specific frameworks used in analysis, reiterated on P7/8
**S15**	**Techniques to enhance trustworthiness** - Techniques to enhance trustworthiness and credibility of data analysis (e.g., member checking, audit trail, triangulation); rationale**	P8: involvement of multiple coders from different disciplines with consensus approach to final coding<br>P9/17: uses of multiple sources of data to triangulate findings, member checking (na)

Results/findings

S16	**Synthesis and interpretation** - Main findings (e.g., interpretations, inferences, and themes); might include development of a theory or model, or integration with prior research or theory	P10-14: outlines description of strategy, BCTs identified at each level and aspects not captured by the taxonomy
S17	**Links to empirical data** - Evidence (e.g., quotes, field notes, text excerpts, photographs) to substantiate analytic findings	P10-14: quotes from documents and interviews<br>Table 2 & 3 outlines BCTs identified<br>Additional file 4 provides evidence (quotes) and descriptive text to substantiate each BCT identified

Discussion

S18	**Integration with prior work, implications, transferability, and contribution(s) to the field -** Short summary of main findings; explanation of how findings and conclusions connect to, support, elaborate on, or challenge conclusions of earlier scholarship; discussion of scope of application/generalizability; identification of unique contribution(s) to scholarship in a discipline or field	P14: summary of main findings<br>P14/15: how findings connect to and elaborate on previous research and unique contribution<br>P15: unique contribution to characterizing organizational components of implementation strategies<br>P16/17: practical implications discusses scope of application and challenges of specification process<br>P18: unique contribution to maximizing existing implementation strategies
S19	**Limitations** - Trustworthiness and limitations of findings	P17/18: limitations of data collection methods, use of triangulation and participants' language to preserve themes that did not fit with framework

Other

S20	**Conflicts of interest** - Potential sources of influence or perceived influence on study conduct and conclusions; how these were managed	P19
S21	**Funding** - Sources of funding and other support; role of funders in data collection, interpretation, and reporting	P19

Reference:

O'Brien BC, Harris IB, Beckman TJ, Reed DA, Cook DA. **Standards for reporting qualitative research: a synthesis of recommendations.** *Academic Medicine*, Vol. 89, No. 9 / Sept 2014
DOI: 10.1097/ACM.0000000000000388
