# Supplementary material for: ‘Around the edges’: using behaviour change techniques to characterise a multilevel implementation strategy for a fall prevention programme
Source: Implement Sci. 2018 Aug 20;13:113. doi: 10.1186/s13012-018-0798-6 (PMC6102850; doi:10.1186/s13012-018-0798-6)
Supplement: Supplementary file 5 — List of BCTs and functions used at the organisational level targeting behaviours ‘supporting the implementation of fall risk assessment clinics’. Table outlining the activities undertaken within each component of the implementation strategy, the BCT coded to that activity and the intervention function(s) it served. (PDF 358 kb) [file 13012_2018_798_MOESM5_ESM.pdf]

Additional File 5

List of BCTs and functions used at the organisational level targeting behaviours 'supporting the implementation of fall risk assessment clinics'

	Component	BCT identified	Function
ORGANISATIONAL LEVEL	**Assembled implementation steering group (SG)** *"of people close enough to the frontline that they knew what was going on but at the same time influential enough to make a difference." (interview)*	Restructuring social environment<br>Credible source	Environmental restructuring<br>Persuasion
	Developed project plan of objectives, deliverables and timelines	Action planning, Goal setting (outcome)	Enablement
	Developed communication plan with list of tasks, target groups, methods and forums for communicating with stakeholders. Included some timelines and assigned responsibilities.	Action planning	Enablement
	Members of SG were involved in selling the clinics to staff being asked to run the new service: *"I tell them it's for an actual screening tool and that they're the decision makers around it, they can decide whether the client needs to be referred on, they can do the plan of care and that client can provide input for what they need. They like that." (interview)*	Framing/reframing,<br>Vicarious consequences (for client)	Persuasion
	Project manager and other SG members involved in "*a round of communications"* targeting different stakeholders: *"it's selling anything like that [the clinic]: [saying to stakeholders] this is fantastic, you can't do without it but it's no trouble [to implement]" (interview)*	Credible source	Persuasion<br>Education
	Monthly meetings to discuss progress, barriers, identified potential solutions and assigned responsibility. The coordinator reported back to the SG and members provided feedback on progress and activities.	Problem solving<br>Feedback on behaviour	Enablement<br>Education
	Central administrative database recording *"activity metrics on numbers referred, waiting times, assessment outcomes, onward referral"*, reviewed at meetings to "*adapt and refine pathway in response to analysis of emerging data". (*document)	Self-monitoring of outcome(s) of behaviour.	Education<br>Enablement
	**Appointed coordinator to act as "a single point of contact"** for referrals and multidisciplinary team (MDT) queries	Restructuring physical environment	Environmental restructuring
	Recruited administrator to manage referrals, appointments for clients and paperwork associated with assessment.	Restructuring social environment<br>Social support (practical)	Environmental restructuring<br>Enablement

	Coordinator (& SG members) identified clinic sites and rooms to hold assessments.	Restructuring physical environment<br>Problem solving	Environmental restructuring<br>Enablement
	**Set up multidisciplinary teams to deliver assessments**		
	Coordinator (& SG members) met heads of discipline to identify staff for teams: *"you identify staff that are suitable and competent to be trained to work within the clinics…/you have to identify people who want to do it" (interview)*	Restructuring social environment	Environmental restructuring

List of BCTs and functions used at the professional level targeting referral to and delivery of fall risk assessment clinic

	Component	BCT identified	Function
PROFESSIONAL LEVEL: MDT	**Coaching MDT**		
	Planned clinics (2 hrs on a set day) with MDT & heads of discipline.	Action planning	Enablement
	Regular contact between MDT & coordinator to answer clinical, administrative & logistical queries.	Social support (unspecified)<br>Social support (practical)	Enablement
	Meetings between MDTs, coordinator & SG members to address problems. Teams told "*its your clinic*" & encouraged to drive decisions (interview)	Problem-solving<br>Framing & reframing	Enablement<br>Persuasion
	Administrator coordinated referrals and appointments, dealt with client queries, sent records and documentation to MDT in advance of clinics and visited clinics to set up administrative processes.	Social support (practical)<br>Instruction on how to perform behaviour	Enablement<br>Training
	**Training MDT**		
	Delivered by coordinator with professional qualification in nursing and "clinical experience in falls" (document)	Credible source	Persuasion
	Group (or one-on-one) training session		
	Instruction on what questions to ask client, assessment sequence, what info to give clients, how to return documentation.	Instruction on how to perform behaviour	Training
	Demonstration of risk assessment by coordinator (e.g. tandem stand test), and online tool to calculate risk score for fractures	Demonstration of behaviour	Training
	MDT members encouraged to use expertise within team, to seek each other's opinion and/or ask colleagues to observe parts of assessment.	Social support (practical)<br>Framing/reframing	Enablement<br>Persuasion

	MDT reassured about ease of conducting assessment, that it takes time to do it properly, scope to use judgement during clinic and are reassured that its ok to ring with queries	Verbal persuasion about capability	Persuasion
	MDT practiced calculating the FRAX score online	Behavioural practice	Training
	Discussed issues such as suitability of space, how best to access equipment and where to locate it.	Problem solving	Enablement
	MDT observed coordinator conducting an assessment with a client		
	Demonstration of risk assessment by coordinator with a client	Demonstration of behaviour	Training
	Debriefing with MDT: coordinator discussed case and the results	Instruction on how to perform behaviour	Training
	Coordinator asked MDT about initial assessments, how they went, and the follow up required by the team.	Problem solving<br>Social support (unspecified)	Enablement<br>Persuasion
	Coordinator supervised members of MDT conducting assessment		
	Coordinator reviewed case and gave feedback on how assessment went, aspects they appeared comfortable with etc. Provided support and encouragement, answered questions & suggested next steps.	Feedback on behaviour<br>Social support (unspecified)	Education<br>Enablement
	MDT visited specialist falls clinic in hospital		
	MDT attend specialist clinic at hospital to "shadow" specialist team & "*see how they work because it is a little bit more detailed"* (interview)	Demonstration of behaviour<br>Credible source, Social comparison<br>Social support (practical)	Modelling<br><br>Enablement
	**Standardised assessment documentation**		
	Standardised assessment form including instructions on what to advise client, what to do on completion & referral options	Adding objects to the environment<br>Prompts and cues	Environmental restructuring<br>Education/Enablement
	Summary of assessment sent with onward referral, included checklist of risks identified, assessments completed & requests for follow-up	Adding objects to the environment<br>Prompts and cues	Environmental restructuring<br>Enablement
	**Equipment**		
	MDTs provided with assessment equipment filing cabinet, scanner	Adding objects to environment	Environmental restructuring
	**Standardised referral form**		

	Provided referrers with standardised referral form which included summary of referral criteria and exclusion criteria	Adding objects to the environment<br>Prompts/cues	Enablement<br>Education
	**Standardised screening tool for PHNs to identify eligible clients**<br>Screening tool added to PHN documentation to identify suitable referrals. This screen was required when requesting equipment: *“any aids or appliances that people are applying for won't be issued unless there's a falls screen” (interview)*	Adding objects to the environment<br>Prompts and cues<br>Behavioural cost	Enablement<br>Environmental restructuring<br>Coercion
	**Information meetings with potential referrers**<br>Meetings to “sell” the service to health professionals in the locality with referral access and share information about referral criteria. Led by coordinator, specialists in gerontology and/or project manager: *“it’s finding the people who already have good relationships.” (interview)*	Credible source<br>(applies across all meetings)	Persuasion
	Meetings with General Practitioners		
	GPs encouraged to refer patients to newly established single point of contact and were told prevention clinics would free up capacity in specialist service so patients would be seen quicker.	Vicarious consequences	Persuasion
	Members of the SG emphasized the benefits of the clinic and minimising the amount of work involved in referral & follow-up*: “You have to say to the GPs ‘this is great but you won’t have to do much.’”*		Persuasion/Education
	Meetings with Advanced Nurse Practitioners (ANPs)		
	ANPs encouraged to contact coordinator to discuss cases and appropriateness of referral	Social support (practical)<br>Feedback on behaviour	Enablement<br>Education
	Meetings with Public Health Nurses (PHNs)		
	Clinics highlighted by mgmt. at quarterly meetings. PHNs who delivered clinics provided feedback on *“how this works, how their patients proceeded to the clinic. So, they [other PHNs] learn from that as well.”*	Credible source<br>Social comparison	Persuasion
	**Promotional Material**		
	Posters provided to health providers and health centres to advertise the service, provide contact details, and summarise the referral criteria	Adding objects to the environment<br>Instruction on how to perform behaviour<br>Prompts and cues	Environmental restructuring<br>Education/Training
	Monthly mail shot to GPs ‘to remind’ them about the service	Prompts and cues	Education

List of BCTs and functions used at the patient level targeting attendance at the risk assessment clinic

	Component	BCT identified	Function
PATIENT LEVEL	**Standardised appointment letter and client information leaflet**		
	Appointment letter notifying client that appointment had been made at clinic. Letter included a list of things the client should bring to the appointment & how to rearrange appointment.	Adding objects to the environment<br>Instruction on how to perform behaviour<br>Imaginary reward	Environmental restructuring<br>Education/Training<br>Persuasion
	Information leaflet sent to clients to invite them to attend the fall risk assessment clinic outlining purpose and benefits of assessment.	Adding objects to environment<br>Instruction on how to perform behaviour<br>Information about health consequences<br>Imaginary reward	Environmental restructuring<br>Education/Training<br>Persuasion
